# CT Findings of Axillary Tuberculosis Lymphadenitis: A Case Detected by Breast Cancer Screening Examination

**DOI:** 10.1155/2016/9016517

**Published:** 2016-06-09

**Authors:** Hiroko Shojaku, Kyo Noguchi, Tetsuya Kamei, Yasuko Tanada, Kouichi Yoshida, Yasuko Adachi, Kazuhiro Matsui

**Affiliations:** ^1^Department of Radiology, University of Toyama, 2630 Suginani, Toyama, Toyama 930-0194, Japan; ^2^Department of Radiology, Saiseikai Takaoka Hospital, 387-1 Futazuka, Takaoka, Toyama 933-8525, Japan; ^3^Department of Surgery, Saiseikai Takaoka Hospital, 387-1 Futazuka, Takaoka, Toyama 933-8525, Japan; ^4^Department of Respiratory Internal Medicine, Saiseikai Takaoka Hospital, 387-1 Futazuka, Takaoka, Toyama 933-8525, Japan; ^5^Department of Pathology, Saiseikai Takaoka Hospital, 387-1 Futazuka, Takaoka, Toyama 933-8525, Japan

## Abstract

We report the first description of CT findings of axillary tuberculous lymphadenitis confirmed by the pathological specimen. The breast cancer screening examination is one of the prime methods of detection of axillary tuberculous lymphadenitis. The most common site of axillary tuberculous lymphadenitis is the deep axilla. Screening mammography often fails to cover the whole axilla. The presence on the contrast-enhanced CT of unilateral multiple circumscribed dense nodes, some of which have large and dotted calcifications, might suggest tuberculous lymphadenitis in axillary region.

## 1. Introduction

Peripheral tuberculous lymphadenitis occurs predominantly in females and develops in people at ages younger than those of other tuberculous conditions [1, 2]. The clinical features and the age distribution between peripheral tuberculous lymphadenitis and breast cancer with axillary metastasis overlap and may initially lead to misdiagnosis [2]. Due to the high prevalence of breast carcinoma and tuberculosis especially in Asia, the coexistence of breast carcinoma and axillary tuberculous lymphadenitis and concomitant tuberculosis and metastasis in axillary lymph nodes has been reported without any radiological examination being performed, so histology and microbiology are essential [3, 4]. The treatment of both diseases is quite different, so it is imperative to perform necessary radiological examinations to expose the possibilities for differential diagnosis.

Although the discovery of axillary tuberculous lymphadenitis has been reported chiefly via mammography [1, 2, 5], there are no reports of the condition being identified by a contrast-enhanced CT [6]. We herein report a case of axillary tuberculous lymphadenitis mainly from the viewpoint of the contrast-enhanced CT findings and their relation to the other radiological findings and pathologic correlations.

## 2. Case Report

A 67-year-old woman presented with a left palpable, fingertip-sized axillary mass that was found by palpitation during breast cancer screening. No breast mass or cervical lymph adenopathy was palpable. The patient was 147 cm in height and weighed 45 kg. Her white blood cell count and C-reactive protein levels were within normal limits.

**Figure 1:**
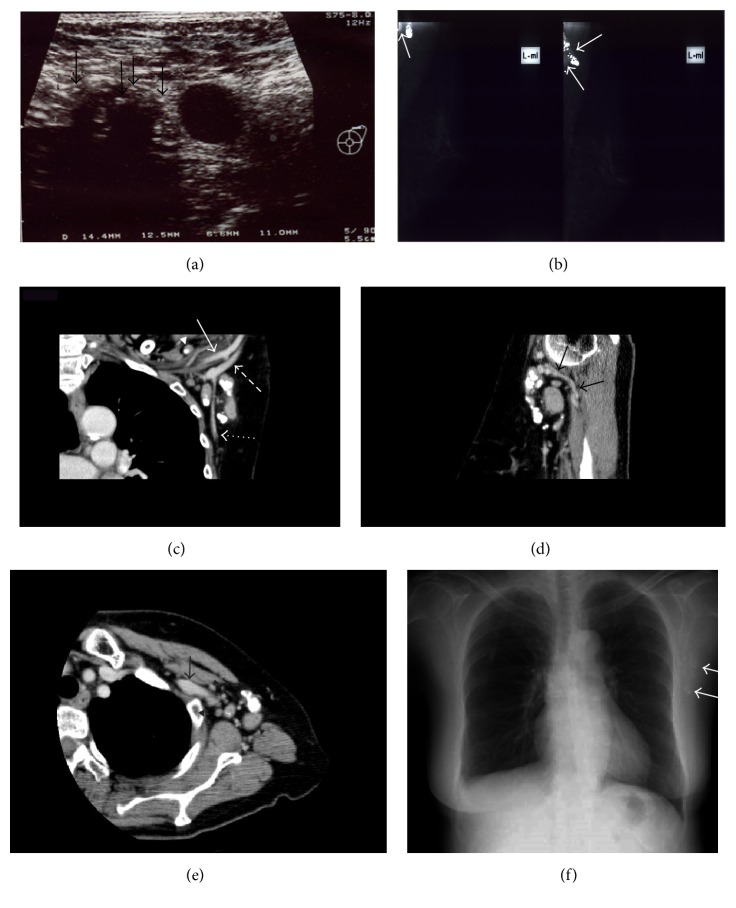
Images from a 67-year-old woman with a left axillary mass. (a) Axillary US shows well-circumscribed markedly hypoechoic ovoid lymph nodes. Every node lacks a hilum. Note the multiple rough calcifications (arrows) within the lymph node. (b) Axillary tail mammographic films show irregularly shaped macrocalcifications and matted, slightly dense homogeneous lymph nodes (arrows). Screening mammographic films show normal findings (not shown). (c) Coronal CT image after administration of intravenous contrast shows the presence of multiple well-circumscribed lymph nodes spread throughout the axillary vein (long dotted line) and dorsal thoracic vein (dotted line) in the left axilla and supraclavicular (arrowhead) and infraclavicular regions. Most nodes have large and dotted calcifications. Arrow indicates axillary artery. (d) Sagittal CT image shows the largest node, which has no calcification itself but is surrounded by nodules rich in calcification. Arrows indicate dorsal thoracic vein. (e) Axial CT image shows the lymph nodes are distributed extending from the axilla to the infraclavicular region around the axillary vein (arrow). (f) Chest radiograph shows clustered calcifications in the left deep lower axilla (arrows).

Screening mammography revealed no abnormalities in the breast or axilla. Ultrasound (US) showed unilateral multiple, markedly hypoechoic, ovoid lymph nodes, some of which had multiple coarse calcifications (Figure 1(a)). We then tried left axillary tail mammography, which revealed irregularly shaped macrocalcifications and dense tissue nodes partially at the corner of the films (Figure 1(b)).

Non-contrast-enhanced CT showed a cluster of 12 well-circumscribed dense nodes spread around the vessels throughout the left deep axilla and supraclavicular and infraclavicular regions. Some of the nodes had large and dotted calcifications. The noncalcified sites of the lymph nodes showed 54–62 Hounsfield units (HU) on the non-contrast-enhanced CT. On contrast-enhanced CT, they appeared to be highly homogenous and showed 98–102 HU. The lymph nodes were partially to completely calcified, and both the calcified and noncalcified nodes were randomly distributed. The largest node measured 3.0 × 2.8 × 1.7 cm (Figures 1(c)–1(e)). The CT images also showed right parabronchial lymph node calcification and small pulmonary nodes in the apex of the right lung, that is, the primary tuberculous complex. A chest radiograph before biopsy showed clustered calcifications in the left deep lower axilla (Figure 1(f)).

**Figure 2:**
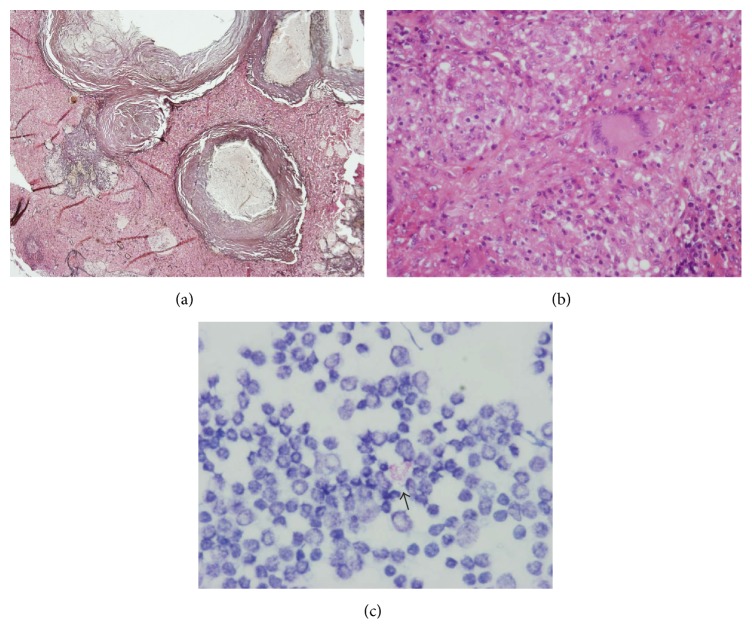
Photomicrography findings. (a) Photomicrograph in low-power view shows caseating necrotizing granulomas with calcification in the central areas (Ag stain, ×40). (b) Photomicrograph shows many necrotizing granulomas that occupy the whole area of the lymph node. Note the Langhans-type multinucleated giant cell (HE stain, ×400). (c) A stamp cytological section stained for acid-fast bacilli reveals Gram-positive rod-shaped bacilli (arrow) (×1000).

Because we could not rule out malignancy as the cause of the pathological nodes, an excisional biopsy was performed. The excised fibroadipose tissue specimen measured 8 × 8 × 1.5 cm and contained several lymph nodes of various fingertip sizes. The histological sections revealed necrotizing granulomas within the architecture of the lymph nodes resulting in the fact that the disease was diagnosed to be active. Calcification was observed in areas of caseating central necrosis (Figures 2(a) and 2(b)). Stamp cytology was performed according to the Ziehl-Neelsen method, which revealed clusters of positive rod-shaped bacilli on the Ziehl-Neelsen-stained slide (Figure 2(c)).

A tuberculin skin test performed after surgery was positive (0 × 0/30 × 28). Smear staining from the bronchial aspiration fluid resulted in a Gaffky scale rating of 0. Culture and PCR of the left axillary lymph nodes and gastric juice were performed, and both were negative. The patient underwent antituberculosis treatment for 9 months. She has remained symptom- and disease-free for over 6 years.

## 3. Discussion

We present the contrast-enhanced CT findings of axillary tuberculous lymphadenitis characterized by unilateral multiple circumscribed well enhanced dense nodes around the vessels in the deep axilla, some of which had large and dotted calcifications. The pathological section showed necrotizing granulomas, which could result from tuberculous lymphadenitis found beyond the proliferative lesions [7]. The contrast-enhanced CT diagnosis of axillary tuberculous lymphadenitis and its differential diagnosis from other disease have not been reported.

To our knowledge, there is only one CT report of the axilla that mentions calcifications, which was of pilomatrix carcinoma. This rare malignant soft-tissue tumor had a well-circumscribed mass with multiple small calcifications and cystic components [8]. The CT diagnosis of axillary tuberculous lymphadenitis and its differential diagnosis from other diseases may be difficult. However, the presence on CT of unilateral multiple circumscribed dense nodes around vessels, some of which have large and dotted calcifications, might suggest axillary tuberculous lymphadenitis. We propose this description as a new CT finding suggestive of axillary tuberculous lymphadenitis because it corresponds to the findings from other modalities.

Calcification in the axillary lymph nodes is uncommon on mammography [9–11] although approximately 5–20% of primary breast carcinomas have mammographically detectable calcifications. In our case, axillary tail mammography showed unilateral irregularly shaped macrocalcifications and dense tissue nodes. Similar findings were reported in 3 of 10 cases in which lymph nodes pathologically confirmed as axillary tuberculous lymphadenopathy showed macrocalcification [2]. Axillary lymphadenitis caused by a prior tuberculous infection may include large, coarse calcifications [5]. Contrastingly, microcalcifications detected on mammography were described in cases of metastasis from breast carcinoma [11], ovarian carcinoma [12], metastatic papillary carcinoma [10], and fat necrosis [9]. Axillary macrocalcification might be helpful in suggesting a diagnosis of tuberculous lymphadenitis.

The US findings in our case showed multiple markedly hypoechoic axillary lymph nodes with multiple coarse calcifications without visualization of a normal central fatty hilum. This US finding was described previously in only one patient who had three nodes of relatively hyperechoic and hypoechoic conglomerate nodes that lacked normal fatty hilum, and no calcification was detected, even though dense calcification was detected on the mammogram [1].

Tuberculosis lesions are histologically characterized as proliferative or exudative. Proliferative lesions are granulomas composed of compact aggregates of epithelioid cells, lymphocytes, and Langhans-type giant cells with variable degrees of central necrosis and relatively few acid-fast bacilli. Exudative lesions consist of an amorphous exudate of mononuclear cells, neutrophils, fibrin, and usually extensive necrotic debris [7]. We showed in the histological section that calcifications were observed in areas of caseating central necrosis, and these calcifications were revealed in every radiological examination on our patient that contained a chest radiograph. The extreme enhancement of the nodes seen on CT in our case reflects the granulomas and aggregate inflammatory cells. From the histopathological viewpoint, our case and the other reported cases of axillary tuberculous lymphadenitis were thought to be of proliferative lesions [1, 2, 4].

Screening mammography often fails to cover the deep axilla, and US for breast cancer screening does not survey the infraclavicular and supraclavicular regions. Associated supraclavicular lesions were reported in 7 of 10 cases of axillary tuberculous lymphadenitis, and all were unilateral [2]. None of the reports on tuberculous lymphadenitis mentioned the infraclavicular region. The axilla and nearby lymph nodes regions can be easily surveyed and objectively viewed with CT, so we can observe the distribution of the lesions and the unique calcification within the nodes, which may help in reaching a diagnosis of axillary tuberculous lymphadenitis.

We described the first enhanced CT findings, to our knowledge, of axillary tuberculous lymphadenitis correlated by other radiologic modalities and pathologic studies. The breast cancer screening examination can be a useful tool for discovering findings of axillary tuberculous lymphadenitis and mammary tuberculosis. We found multiple unilateral circumscribed and well-enhanced dense nodes around the vessels in the deep axilla, some of which had large and dotted calcifications indicative of tuberculous lymphadenitis.

In this case, the axillary lymphadenitis was diagnosed as the active disease because the pathological specimen by the biopsy of the node shows the necrotizing granulomas. Her axillary lymph nodes were calcified and homogenously enhanced in the contrast CT. So, we think that the calcification of the axillary nodes might be one of characteristic findings on contrast CT in tuberculous lymphadenitis, but it might not be helpful for diagnosing the activity of the disease. Further studies are necessary to evaluate this possibility.

Early diagnosis and treatment of active tuberculosis decrease the opportunities for the spread of the disease in the community. Thus, physicians should familiarize themselves with the radiological findings associated with axillary tuberculous lymphadenitis.

## 4. Conclusion

The breast cancer screening examination is one of the prime methods of detection of axillary tuberculous lymphadenitis. On US the notice of coarse calcification within the nodes helps in the suspicion of the disease. Further examination on the contrast-enhanced CT and the presence of unilateral multiple circumscribed dense nodes, some of which have large and dotted calcifications, might suggest tuberculous lymphadenitis in axillary region. Early diagnosis and treatment of tuberculosis decrease the opportunities for the spread of the disease in the community.
